# Study of a set of reading precursors among Chilean children with Down syndrome

**DOI:** 10.3389/fpsyg.2023.1090710

**Published:** 2023-02-06

**Authors:** Paulina S. Arango, Jose P. Escobar, Pelusa Orellana, Andrés Aparicio, Katherine Strasser, Ricardo Rosas, Marcela Tenorio

**Affiliations:** ^1^Millennium Institute for Care Research (MICARE), Santiago, Chile; ^2^Universidad de los Andes, Chile, Santiago, Chile; ^3^Pontificia Universidad Católica de Chile, Santiago, Chile

**Keywords:** reading, reading precursors, Spanish, Down syndrome, phonological awareness

## Abstract

Learning to read for children with Down syndrome is relevant because of the impact this ability has on learning and the development of autonomy. Previous research has described reading development in this population, but it is not clear if the process and precursors are the same in a transparent language like Spanish. This study explores performance in a set of precursors (phonological awareness, visual recognition, vocabulary, letter knowledge and verbal reasoning) in 42 children with Down syndrome between 6:0 and 10:11 years. We hypothesized that the participants would have a lower performance than previously reported with children with typical development, particularly in tasks of phonological awareness, because the method for reading instruction in Chile with this population is usually the global method. Our results show that the precursors improve with age, that there are differences in performance between the skills assessed, and the ceiling effect was not observed as would be expected for children with typical development for the abilities assessed at these ages, which suggests that in the children assessed the precursors are not consolidated at these ages. These results suggest that the stimulation of phonological awareness and other reading precursors in children with Down syndrome is important for reading development.

## Introduction

Reading is a necessary skill in the modern world, since it provides access to learning, facilitates the development of functional skills and drives social participation (Cologon, 2013). There are various theories about how reading ability development occurs, and although none is accepted as the only one, there are various common factors among them. In most theories it is proposed that reading depends on mental and linguistic sub-skills called precursors, which shape the system as a gestalt (Coltheart, 2006). The evidence for precursors’ prediction of reading skill is incontrovertible (Adams, 1990), and it explains reading ability variations during typical development (National Reading Panel (US), National Institute of Child Health and Human Development (US), 2000; Torgerson et al., 2006). Some of the relevant reading precursors according to the literature are phonological awareness, decoding, visual recognition of words, language comprehension, and vocabulary (Scarborough, 2002). Studies among Spanish-speaking children show that these precursors are consolidated around the end of preschool education, between the ages of 4 and 6 years (Herrera and Defior, 2005; Vieiro-Iglesias et al., 2015; Morales et al., 2020). Therefore, formal reading instruction begins with these precursors already being consolidated, and by the end of second grade children are already expected to be competent readers.

Unlike the knowledge gained about reading development in typical development, there is little evidence about reading precursors’ development in the presence of Down Syndrome, a congenital condition with high likelihood for intellectual disability (Sherman et al., 2007; de Graaf et al., 2016). The gap increases when considering native Spanish speakers.

Spanish is a Romance language and is the second most widely spoken language in the world (Eberhard et al., 2022). It is composed of 29 graphemes: the 26 letters of the English alphabet plus the letter ñ and the digraphs ch and ll (Goikoetxea, 2006). At the phonetic level there are 26 phonemes, of which 5 are vowels and the rest consonants. A syllable in Spanish has a maximum of 5 phonemes, with a maximum of two initial consonants (Defior and Serrano, 2014). The most frequent syllable structure is CV followed by CVC. Spanish has an alphabetic phonological orthographic system, and although there are geographical phonological differences, they do not generate comprehension problems between speakers (Defior and Serrano, 2014). The correspondence between graphemes and phonemes is almost biunivocal. Therefore, Spanish is considered a language with a transparent orthographic system due to the high grapheme-phoneme correspondence (Bravo-Valdivieso and Escobar, 2014). This has practical implications for learning to read. For example, children learn to read faster than in opaque orthographic systems (Seymour et al., 2003), preferably through syllabic-phonological methods. Reading is explicitly taught in the first year of basic education, around age 6, and decoding is typically consolidated during the second year of instruction (Defior and Serrano, 2014). Additionally, their orthographic transparency qualities impact the role and relevance of precursor skills. For example, although phonological awareness is relevant and emerges around the age of 4 (Acevedo, 1993), in the initial stages of reading formation it yields its predictive power in favor of variables more related to reading fluency, such as rapid automatized naming (Defior, 2008). It is also the case that, in Spanish as in other shallows orthographies, students acquire orthographic knowledge earlier, which they use to read words (Share, 1995; Goikoetxea, 2006).

It is important to note that education in Chile is compulsory from the first year of basic education, which children enter at age 6, to the last year of secondary education. Regarding preschool education, data from the Ministry of Education indicate that approximately 50% of Chilean children between the ages of 4 and 5 attend kindergarten, including children with disabilities (Subsecretaría de Educación Parvularia, G. de C, 2021). As in other countries, formal reading instruction begins in first grade, although the reading precursors are worked on in the preschool years (Subsecretaría de Educación Parvularia, G. de C, 2018). It is important to note that Chile has a unified national curriculum that all schools must follow regardless of whether they are regular or special schools, so every child in the country receives the same amount of reading instruction.

The present study intends to provide knowledge *via* analyzing the development traits of a set of reading precursors among a group of Chilean children with Down Syndrome. We follow the model of Scarborough (2002) who stated that competent reading has fluid and coordinated integration of two dimensions, namely recognition of written words and language comprehension. Within the recognition of written words, we can include phonological awareness, decoding and the visual recognition of words. Language comprehension includes conceptual knowledge as well as knowledge about linguistic structures, vocabulary, verbal reasoning, comprehension of figurative language, literary genres and their characteristics (Scarborough, 2002).

Considering that there is agreement about how the central reading precursors are phonological awareness, decoding, visual recognition of words, vocabulary and verbal reasoning, the study is centered on exploring these dimensions (National Reading Panel (US), National Institute of Child Health and Human Development (US), 2000).

### Phonological awareness

This is a metalinguistic skill allowing its users to perceive and manipulate language units, and is indispensable for acquiring the alphabetical principle, that is, the notion that in alphabetic spellings such as Spanish, the written symbols represent sounds, and not other units such as ideas or words (Bravo Valdivieso, 2002). There is evidence that without this skill, it is not possible to move into more complex areas of learning to read such as inferential comprehension (Bianco et al., 2012), which is similar in languages with different levels of spelling transparency (Caravolas et al., 2005).

Various tasks have been used to evaluate phonological awareness, with the most common ones being segmentation of syllables and phonemes, rhyme recognition, and initial and final sounds and syllables (Goswami and Bryant, 2016). There is evidence suggesting that in the presence of typical development, Spanish speakers can achieve competence in syllable segmentation between the ages of 4 and 5 years (Vieiro-Iglesias et al., 2015; Morales et al., 2020) and for syllable discrimination between ages 5 and 6 years (Aguilar Villagrán et al., 2011).

Given the recognized importance of phonological awareness as a reading precursor, it was ultimately incorporated as a relevant variable to understand reading development in Down Syndrome. Pioneering studies marked the area, particularly the work of Cossu and Marshall (1990) who stated that efficient oral reading in fact did not necessarily require a good level of phonological awareness. This affirmation assumed, among other things, the idea that children with Down syndrome learned to read by methods which were qualitatively different from children with typical development. Their evidence came from observing children with Down syndrome who achieved oral reading, but had very low scores in phonological awareness tests, which presented a disassociation of domains as something peculiar to their genetic condition.

Later studies (Cupples and Iacono, 2000; Fletcher and Buckley, 2002; van Bysterveldt and Gillon, 2014) have debated this finding by showing that the exploration of phonological awareness in Down syndrome faces a sizable challenge, namely the adaptation of paradigms to get the participants to understand complex instructions. Therefore, more than being a group which cannot achieve phonological awareness, it is a problem of access to reliable information due to shortcomings in the measuring instruments. With adaptations to the experimental paradigms, it has been shown that children with Down syndrome substantially improve their performance on phonological awareness tasks over time and with systematic school instruction (Kay-Raining Bird et al., 2000), although it is not yet clear at what age this achievement can be expected. Predictive relations have also been documented between phonological awareness and working memory with reading performance in Down syndrome (van Bysterveldt and Gillon, 2014).

Research has shown that in people with Down syndrome, phonological awareness skills are compromised at different levels depending on the complexity of the task. In this sense, complex skills such as blending and segmenting sounds present low performance (Lemons and Fuchs, 2010a), while performance improves in basic tasks such as initial phoneme identification, although comparatively still below the performance of peers with typical development (Kalaycı and Diken, 2022). Rhyme detection tasks have also been found to be challenging for this population (Verucci et al., 2006; Næss, 2016), although there are also studies reporting rather moderate compromises (Kalaycı and Diken, 2022). Regarding syllable segmentation, the results are not conclusive, but with a tendency for these skills to be compromised (Verucci et al., 2006). It is reported that within the more complex phonological skills, children with Down syndrome present more difficulties with syllable segmentation compared to the blending tasks (van Bysterveldt and Gillon, 2014; Kalaycı and Diken, 2022). For other authors, however, the trade-offs are rather moderate. For example, Acarlar et al. (2002) in a sample of Turkish children, report that syllable segmentation is the easiest phonological task for this group. They mention that it is possible that the orthographic qualities of Turkish may explain this effect since Turkish words have short syllables, something similar with Spanish (Defior and Serrano, 2014). Results of the studies of the phonological strengths and weaknesses of people with Down syndrome are relevant because they provide clear perspectives for the reading interventions. For example, they suggest the need for a clear knowledge about the individual’s phonological abilities in order to provide the necessary supports (Lemons, 2015; Hessling Prahl et al., 2022), which in turn impacts on word reading skills (van Bysterveldt and Gillon, 2014).

### Letter knowledge

This consists of applying knowledge about the relations between letters and their sounds to pronounce the words and assign them meaning, and is a precursor of decoding (Beck and Juel, 1995). Visual recognition of words includes synthesizing phonemes or syllables to read larger units and with an associated meaning. Evaluation of letter knowledge is usually done *via* exploring knowledge of graphemes or by knowledge of letters’ names.

Identifying letter sounds and names has been consistently acknowledged as an important predictor of reading (Adams, 1990; Whitehurst and Lonigan, 1998). Letter-sound knowledge facilitates the acquisition of the alphabetic principle, which in turn, helps children decode. Many studies also support the importance of explicitly teaching letter-sound correspondence to facilitate reading development (Ehri et al., 2001; Dehaene and Cohen, 2011; Solheim et al., 2018).

Evidence of interventions to enhance letter identification skills among children with Down Syndrome have yielded significant improvement. For example, bringing children’s attention to printed letter features and phonemes during shared reading facilitated letter knowledge (Van Bysterveldt et al., 2007). Just like typically developing readers, children with Down Syndrome benefit from explicit, systematic, and supplemental instruction on code-related skills such as letter identification (King, 2020; King et al., 2022).

Some authors have described decoding as a relative weakness in children with Down Syndrome, as opposed to visual recognition of words (Verucci et al., 2006; Hulme et al., 2012), while other researchers have not found differences in these skills between children with typical development and Down syndrome on non-timed tasks. This would indicate that the problems lie in processing speed rather than in decoding (Snowling et al., 2002). It should be mentioned that decoding skills’ development among children with Down syndrome has seen little study, which some authors associate with the difficulties of evaluation with tasks designed for children with typical development (Næss et al., 2012).

### Visual recognition of words

One of the most influential models for explaining word reading is the double-route model (Coltheart, 2006). This model states that words can be read by a phonological pathway, when they are new or infrequent words, or else by a visual or lexical route, which is used for reading familiar or frequent words. Readers with more reading experience achieve quicker visual recognition of words and automate this process to consolidate it, when they are native Spanish speakers, around age 6.

Visual recognition of words is a skill which helps free up cognitive resources originally oriented towards decoding words, which can now be redirected towards processes related to reading comprehension (Mimeau et al., 2018). Among people with Down Syndrome, it has been found that word reading is their main reading strength, leading to a focus on intentional learning of word reading from global methods (Troncoso and Flórez, 2011). The studies show that the reading profile of people with Down syndrome presents a discrepancy between word reading level and reading comprehension (Næss et al., 2012), compared with the profile of reading comprehension problems in a typical population. However, it is possible that this profile is not present in a Latin American context, where research has shown that children with Down syndrome have difficulties both in word reading skills and reading comprehension (Hernández Salazar and Talou, 2005).

### Vocabulary

Vocabulary is one of the best predictors of reading comprehension once decoding processes have become automatic. In fact, vocabulary and comprehension have a bidirectional relation that increases over time (Torgesen et al., 1997; Tannenbaum et al., 2009; Quinn et al., 2015). Children’s vocabulary develops through social verbal interactions prior to learning how to read, and the amount of vocabulary knowledge is strongly associated with socioeconomic background (Hoff, 2003). Children’s vocabulary in the early years accounts for a significant amount of variance in reading comprehension, both from a linguistic and cognitive perspective (Duff et al., 2015).

The bidirectional relation between vocabulary knowledge and comprehension can be explained by the fact that understanding the meaning of a word allows a reader to better understand a passage and, as readers become better comprehenders, their vocabulary also increases. At the same time, one can infer that vocabulary and comprehension also contribute to conceptual knowledge, and therefore are strongly correlated (Rupley, 2012). This relation can also be explained if vocabulary and comprehension are part of what is defined as verbal aptitude, so that students who know more about words and concepts are better at building meaning from text (Anderson and Freebody, 1981). From a pedagogical perspective, the bidirectional relation is likely to have more implications for the development of both comprehension and vocabulary.

Studies of the relation between vocabulary and reading have received attention for both children with typical development and with Down syndrome (Kay-Raining Bird et al., 2000) and are often studied *via* exploring receptive vocabulary. Evidence suggests that this skill is consolidated around ages 4 or 5 in children with typical development (Cáceres Zuñiga, 2018).

Although most studies in Down syndrome have been done with Anglophone populations, a significant advance has arisen from knowledge about vocabulary in this group following the Spanish standardization of the MacArthur-Bates Communicative Development Inventories (Mariscal et al., 2007; Galeote et al., 2011; Checa et al., 2016).

Regarding the relation between vocabulary and reading abilities in children with Down syndrome, previous studies have shown strong correlations between these abilities, which tend to be higher in the children with Down syndrome, when compared to children with typical development. For example, Nash and Heath (2011) found stronger correlations between vocabulary and reading comprehension in children with Down syndrome than in those with typical development. Also, in a longitudinal study, Hulme et al. (2012) found that vocabulary was a strong predictor of reading performance both for children with Down syndrome and in children with typical development, but it was a stronger predictor for the former than for the latter.

### Verbal reasoning

This is the capacity to process and use orally presented information and properly integrate it to understand its meaning and manipulate verbal information (Language and Reading Research Consortium, 2017). The influence of verbal abilities such as oral comprehension and verbal working memory on reading development is documented among children with typical development (Lervåg et al., 2018), but there are few studies in Down syndrome (Boudreau, 2002; Roch et al., 2015).

Oral comprehension level tends to be considered a good indicator of verbal reasoning, achieving a good performance level between 4 and 5 years (Florit et al., 2009). Prior studies have shown that this dimension is a weakness for people with Down Syndrome, characterized by difficulties in production and syntactic comprehension (Boudreau and Chapman, 2000), but there are no available studies exploring at which age one can expect performance relatively similar to that observed in typical development when reaching a competent level.

In general terms, there is little literature exploring reading precursors in children with Down syndrome who are native Spanish speakers. There are some studies which have shown the extended belief that for children with Down syndrome reading is equivalent to decoding, without incorporating other predictive dimensions of comprehension, which is not supported by available evidence (Cupples and Iacono, 2002; Jiménez and Flórez-Romero, 2013; de la Cruz Paulino, 2017).

The incorporation of the global method – which emphasizes the reading of words as a Gestalt and not the teaching of the sub-lexical units of language such as sounds and morphemes – dominated methods for teaching reading to children with intellectual disabilities in general, and Down syndrome in particular, for over 30 years (Troncoso and Flórez, 2011). Particularly in Chile, this is still the officially recommended method to teach reading to this population group (Ministerio de Educación, 2008) and is thus the method adopted across the board in special schools and civil society foundations which offer alternative accompaniment to the population.

However, studies have shown that systematic and explicit teaching of the association between letters and their sounds is related with improvements in this population (Lemons and Fuchs, 2010b), meaning that the global method is insufficient (Cologon et al., 2011). Studies of reading skill development require an exploration which responds to the complexity of the process and which, in the case of children with Down syndrome who tend to need help, goes beyond decoding, and allows us to understand the strong and weak points of the process for more efficient support and accompaniment.

This exploratory study was aimed to analyze a set of reading precursors in children with Down syndrome, with a hypothesis that differentiated development levels will be observed compared to those previously reported in the literature about typical development for Spanish speaking children in these precursors, with phonological awareness being the dimension where higher differences are expected, given the lack of attention which it receives under the reigning instructional model for children with Down syndrome within the country, the global teaching method.

## Methods

This study follows a within-subject design, based on the analysis of cross-sectional measures of performance on tasks exploring reading precursors among a group of school-age Chilean children.

### Participants

The study included 42 Chilean children with Down syndrome (21 girls and 21 boys) between 6:0 and 10:11 years (*M = 7.98, SD = 1.44*). Twenty-three participants attended special schools and 19 attended regular schools. The entire sample received educational and learning accompaniment outside of school hours *via* special institutions to support people with Down syndrome. All the schools of the participants stated that they use the global method as the main reading instruction method, and that they follow the indications of the national curriculum. For the analysis, the sample was divided into three age groups: 6:0–7:11 years (*n* = 17), 8:0–9:11 years (*n* = 17) and 10:0–10:11 years (*n* = 8). We decided to make this division by age groups since reading development is related to both age and grade level, and these are the ages that typically correspond to grades 2, 3, and 4 in Chile.

Inclusion criteria were: (1) having confirmed Down syndrome with a karyotype, (2) receiving reading instruction with the global method, (3) presenting appropriate functioning in sensory systems or adequate correction with glasses or hearing aids, (4) having authorization from their legal guardians by signing informed consent and (5) being able to provide consent with a witness for their participation. The exclusion criteria were: (1) having an uncorrected sensory difficulty, and (2) having undergone any type of surgical intervention and/or hospitalization during the month prior to the reading skill evaluation.

### Instruments

We adopted a strategy of evaluation which has been applied in prior studies done with children with Down syndrome who were native English speakers, where paradigms had to be adapted (e.g., van Bysterveldt and Gillon, 2014). Experimental evaluation tests were applied for reading precursors whose validity is documented in previously published studies. This decision is due to the lack of packaged exploration instruments for reading precursors with proper psychometric evidence for a Spanish-speaking Down syndrome population. While there is an extant Spanish version of the MacArthur-Bates Communicative Development Inventories which has been tested on children with Down syndrome in Spain, and which explores vocabulary (Galeote et al., 2012), given the discrepancies between the Spanish used in Spain and Chile, it is not recommendable to use it in our context. Along with this, the age range which is the target for this test (8–30 months) is not the age we wanted to assess in the present study.

The evaluation of phonological awareness included syllable discrimination and segmentation tasks. For decoding, we considered grapheme-phoneme recognition tasks and letter name knowledge. We also used tasks to explore visual recognition, receptive vocabulary, and verbal reasoning. Table 1 presents task descriptions, their variable of interest, and examples of studies which have used the paradigm and offered validity and reliability evidence *via* internal consistency with Cronbach’s Alpha.

**Table 1:** Description of experimental paradigms.

General dimension	Subdimension	Paradigm	Description	Reference studies	Cronbach’s alpha
Written word recognition					
Phonological awareness		Syllable auditory discrimination	The child repeatedly listens to a syllable, e.g., /do/, and must touch the screen when they hear a different syllable, e.g., /ma/. Items are calibrated in a progressively more complex sequence determined by syllables’ phonetic similarity.	Rosas et al. (2011, 2017); Rosas (2013); Abello et al. (2014); Cadavid-Ruiz et al. (2016)	0.694
		Final syllable discrimination	Three images of objects are presented. The child hears the name of each object presented and chooses the one with a different final syllable. Information presentation is auditory, without any input from written words.	Rosas (2013); Abello et al. (2014); Escobar and Meneses (2014); Cadavid-Ruiz et al. (2016); Rosas et al. (2017)	0.722
		Syllable segmentation	The child hears a word and must segment it into syllables *via* a drum which is used by touching a screen. The items were calibrated for progressively rising difficulty according to the words’ extension and syllabic structure.	Rosas (2013); Cadavid-Ruiz et al. (2016)	0.782
Letter knowledge		Grapheme recognition	A letter graph is presented, and the child must choose the representation for the sound of graph they see between four alternatives. The test is calibrated in progressively rising difficulty determined by the order in which letters are taught in a Chilean school context: first vowels, then consonants.	Rosas (2013); Cadavid-Ruiz et al. (2016)	0.768
		Knowledge of letters’ names	The child hears the name of a letter and must pick the right choice from four possible graphs. We used the same items from the grapheme recognition task.	Ricci (2011); Rosas (2013); Cadavid-Ruiz et al. (2016); Rosas et al. (2017)	0.791
Visual recognition		Word Reading	The participant must read increasingly complex words out loud, as they appear on screen. Complexity is determined by the words’ length and syllabic structure. Reading precision is scored.	Rosas et al. (2011); Rosas (2013)	0.897
Language comprehension					
Vocabulary		Vocabulary	Four images are presented, and the evaluator says a word out loud. The child must indicate which image corresponds to the word they heard. For instance, the evaluator says “dog” and the child must choose between a dog, a cat, a pear, and a moon. The test is calibrated for increasing complexity determined by words’ frequency and length. The key variable is precision.	Dunn and Dunn (1997); Strasser et al. (2010)	0.843
Verbal reasoning		Oral Comprehension	The evaluator says a phrase and the child must choose which of three images best represents it. For instance, the evaluator says: “The cat sleeps.” Three images appear: a dog sleeping, a cat playing, and a cat sleeping. The key variable is precision.	Rosas et al. (2011); Rosas (2013); Cadavid-Ruiz et al. (2016)	0.675

### Procedure

Procedures incorporated in this study were supervised by the Ethics Committee at the hosting University.

Children with Down syndrome were invited *via* six support organizations located within the urban area of Santiago, Chile. Parents signed an informed consent authorizing their child’s participation, and the children themselves provided verbal assent.

Evaluations were done by a research assistant with postgraduate neuropsychology training. Tests were applied following a standardized protocol, always in the same order, with an average time of 30 min. The children were evaluated in the organizations they attend or in appropriate spaces at the University. They were accompanied by their parents in all cases and were able to ask to stop or receive help whenever they required. Qualitative performance reports were delivered to each family, accompanied by recommendations for stimulating reading development and a users’ manual for La Mesita, a tablet-based game designed to promote reading development in children with intellectual disability (Tenorio, 2016).

### Data analysis

Data analysis was done considering three precursors associated with visual word recognition according to the model from Scarborough (2002): phonological awareness, decoding and visual recognition. We also analyzed two precursors associated with language comprehension under this model: vocabulary and verbal reasoning.

To supply reliability and validity evidence for the experimental tests, we followed recommendations from the international measurement quality standards for psychology and education (American Educational Research Association, American Psychological Association and National Council on Measurement in Education, 2014). This offered reliability evidence analysis *via* internal structure exploration using Cronbach’s Alpha. Validity evidence is based on content study and paradigm implementation with broad prior use.

We present descriptive data with the means and standard deviations from the sample and consider an analysis of simple correlations with age as a variable, and bivariate Pearson correlations controlled by participants’ age in years. To analyze achievement differences between precursors, we compared performance medians for each age group using one-way ANOVAs for the variables of interest. For each comparison we tested the homoscedasticity with Bartlett’s test. Post-hoc comparisons with Holm-adjusted *p*-values were used to study the difference points.

All analyses were performed in R 4.2.1 (R Core Team, 2022) with packages stats (R Core Team, 2022), psych (Revelle, 2020) and tidyverse (Wickham et al., 2019).

## Results

Preliminary data inspection shows variance, without any floor effect, a commonly observed effect when evaluating children with Down syndrome (Kennedy and Flynn, 2003). For all variables we observe a trend towards improved performance as ages rose (Table 2).

**Table 2:** Descriptive statistics for reading precursors by age group.

		6:0–7:11 M(SD)	8:0–9:11 M(SD)	10:0–10:11 M(SD)
**Recognition of written words**				
Phonological awareness	SAD	0.39 (0.65)	1.15 (1.63)	0.88 (1.46)
	FSD	1.00 (1.30)	1.88 (1.09)	2.25 (1.17)
	SS	0.87 (0.92)	2.50 (2.25)	3.25 (1.49)
Letter Knowledge	GR	2.67 (1.23)	4.81 (2.11)	4.00 (2.27)
	KLN	3.07 (1.79)	4.25 (2.05)	4.13 (2.48)
	WR	2.57 (1.91)	4.43 (0.65)	4.57 (0.79)
**Language comprehension**				
Vocabulary	Voc	5.33 (2.47)	8.19 (1.76)	8,00 (1.93)
Verbal reasoning	OC	2.29 (1.38)	3.50 (1.27)	3.88 (0.84)

SAD= syllable auditory discrimination, max = 6; FSD = Final syllable discrimination, max = 6; SS = Syllable segmentation, max = 6; GR = Grapheme recognition, max = 7; KLN = knowledge of letters’ names, max = 7; WR = Word reading, max = 12; Voc = Vocabulary, max = 10; OC = Oral comprehension, max = 5.

The simple correlations matrix shows a positive, strong, and significant correlation between age of the participant in years and vocabulary, oral comprehension, final syllable discrimination, word recognition and syllable segmentation. No correlation was found for age and grapheme recognition, knowledge of letters’ names and syllable auditory discrimination (Table 3).

**Table 3:** Simple correlations between variables of interest and age in years.

	SAD	FSD	SS	GR	KLN	WR	Voc	OC	Age
SAD	1	0.044	0.332*	0.281	0.244	0.084	0.222	0.169	0.121
FSD		1	0.23	0.304	0.375	0.478**	0.422	0.169	0.363*
SS			1	0.565**	0.533*	0.493*	0.593**	0.383	0.399**
GR				1	0.765***	0.567***	0.769***	0.550**	0.303
KLN					1	0.518***	0.664***	0.391	0.184
WR						1	0.802***	0.301	0.436**
Voc							1	0.426**	0.393*
OC								1	0.491**
Age									1

***p < 0.001; **p < 0.01; * *p* < 0.05. SAD = syllable auditory discrimination; FSD = Final syllable discrimination; SS = Syllable segmentation; GR = Grapheme recognition; KLN = knowledge of letters’ names; WR = Word reading; Voc = Vocabulary; OC = Oral comprehension.

When analyzing the partial correlations matrix (Table 4), there is a notable presence of positive, strong, and significant relations between vocabulary and the other variables explored, apart from oral comprehension and syllable discrimination. There are also positive, strong, and significant correlations between word recognition and syllable segmentation, grapheme recognition, vocabulary, and knowledge of letter’s names. There is another similar pattern in the grapheme recognition and knowledge of letter’s names variables.

**Table 4:** Partial correlations between variables of interest controlled by age in years.

	SAD	FSD	SS	GR	KLN	WR	Voc	OC
SAD	1	0	0.312*	0.258	0.227	0.035	0.191	0.263
FSD		1	0.1	0.218	0.336	0.382*	0.326	−0.012
SS			1	0.508***	0.510***	0.387*	0.517***	0.235
GR				1	0.757***	0.507***	0.742***	0.483*
KLN					1	0.495***	0.654***	0.351
WR						1	0.762***	0.112
Voc							1	0.292
OC								1

*** *p* < 0.001; **p < 0.01; * *p* < 0.05. SAD = syllable auditory discrimination; FSD = Final syllable discrimination; SS = Syllable segmentation; GR = Grapheme recognition; KLN = knowledge of letters’ names; WR = Word reading; Voc = Vocabulary; OC = Oral comprehension.

In the comparison between age groups there are several statistically significant differences in favor of older children with small effect sizes (Cohen, 1992; Dunst and Hamby, 2012; Bakker et al., 2019). Regarding phonological awareness exploration, there are significant differences by age group for final syllable discrimination (*F* = 3.41, *p* < 0.05, η^2^ = 0.166) and syllable segmentation (*F* = 10.22, *p* < 0.001, η^2^ = 0.258) but not syllable auditory discrimination (*F* = 1.44, *p* = 0.269, η^2^ = 0.074). In letter knowledge, while there is a statistically significant difference for grapheme recognition (*F* = 5.23, *p* < 0.01, η^2^ = 0.230), there is no such difference in knowledge of letter names (*F* = 1.45, *p* = 0.249, η^2^ = 0.076). In visual word recognition, there are significant differences in word reading (*F* = 6.16, *p* < 0.05, η^2^ = 0.350). There are also significant differences for vocabulary (*F* = 8.23, *p* < 0.001, η^2^ = 0.320) and oral comprehension (*F* = 5.41, *p* < 0.01, η^2^ = 0.242).

The post-hoc tests show that all variables appear to stabilize between the second age group (age 8 to 9) and the third one (10 years), as no significant differences are found between these two older groups (Table 5). Regarding the comparisons between the first age group (6 and 7 years) to the second one (age 8 and 9) and between the first and the third one (age 10) there are three patterns: a significant change between the two younger groups that stabilizes afterwards (Grapheme recognition: *F* = 4.027, *p* < 0.01), a significant change when comparing the youngest and oldest groups (Final syllable discrimination: *F* = 5.051, *p* < 0.05), and significant differences between both the two younger groups and the youngest and oldest groups (Syllable segmentation: *F* = 7.161, *p* < 0.05 and *F* = 17.075, *p* < 0.01; Word recognition: *F* = 11.876, *p* < 0.01 and *F* = 11.460, *p* < 0.01; Vocabulary: *F* = 13.380, *p* < 0.001 and *F* = 6.998, *p* < 0.05; and Oral comprehension: *F* = 6.310, *p* < 0.05 and *F* = 8.650, *p* < 0.01). No ceiling effect was observed in any variable.

**Table 5:** *Post-hoc* analyses for the variables with significant differences by age group.

Variables / comparisons		Group 1 – Group 2		Group 1 – Group 3		Group 2 – Group 3	
		F	*p*	F	*p*	F	*p*
Phonological awareness	FSD	4.027	0.055*	5.051	0.036*	0.606	0.445
	SS	7.161	0.014*	17.075	0.002*	0.948	0.342
Letter knowledge	GR	11.778	0.002*	3.397	0.079	0.756	0.394
Visual recognition	WR	11.876	0.003*	11.460	0.003*	0.173	0.686
Vocabulary	Voc	13.880	0.001*	6.998	0.015*	0.057	0.814
Verbal reasoning	OC	6.310	0.018*	8.650	0.008*	0.571	0.458

Group 1: 6–7 years; Group 2: 8–9 years; Group 3: 10 years; SD = syllable discrimination; FSD = Final syllable discrimination; SS = Syllable segmentation; CG = Grapheme recognition; KLN = knowledge of letters’ names; WR = Word reading; Voc = Vocabulary; OC = Oral comprehension.

## Discussion

This study explored the performance of a group of school aged children with Down syndrome who received reading instruction with the global teaching method, on a set of reading precursors. We analyzed the variables which took part in emergent reading among this population, to reflect on the transformations which should be considered when designing educational programs oriented towards reading instruction for children with Down syndrome.

The first notable result is that none of the variables explored showed a ceiling effect for any of the three age groups which suggests that for the children in our sample, reading precursors are not yet consolidated at age 10. The evidence from previous studies shows that in children with typical development, all the precursors explored in our study are consolidated within the first age range explored in this study (6:0 to 7:11 years) (Rosas et al., 2011; Bravo-Valdivieso and Escobar, 2014; Escobar and Meneses, 2014). Considering the delay normally observed in Down syndrome, it should lie within the higher age range for this group (10:0–10:11 years). This finding is in line with our general hypothesis and provides evidence for a general delay of reading precursors within the evaluated group, indicating that children with Down syndrome need even more time to consolidate these skills and thereby, move forward in the formal process of learning to read.

In almost all the variables explored we also observed progressive improvement in performance, which shows that children with Down syndrome can learn these abilities, as previous studies have shown (Boudreau, 2002; Goetz et al., 2008; Baylis and Snowling, 2011). Although in this study we used experimental tasks and did not assess abilities through standardized tests to measure the variables evaluated, there is ample evidence about the ages at which Spanish-speaking children develop the reading precursors explored in this study (Seymour et al., 2003; Ziegler and Goswami, 2005; Míguez-Álvarez et al., 2022). This evidence allows us to suggest that the achievement level observed did not reach the same level that should be expected for children with typical development according to the literature, and also that it was not the same expected for children with Down syndrome who received instruction focused on stimulating phonological awareness, according to available reports (Baylis and Snowling, 2011).

One notable result from this study is the qualitative leap observed in performance quality at age 8 for variables where age progression was significant. We found significant differences between the first age group (ages 6 and 7) and the other two age groups in the syllable segmentation, word reading, vocabulary, and oral comprehension tasks. In the grapheme recognition task, there was a significant difference between the first and second (ages 8 and 9) age groups, and in the final syllable discrimination task a significant difference was found between the first and the older age groups. The differences found in the overall mean comparison analyses in these variables showed small effect sizes. In these variables our results suggest that children with Down syndrome have a significant performance improvement after age 7, according to the differences found between the three group ages compared in this study. It is complex to offer a conclusive explanation for this finding given the scope of this study, but we may hypothesize that this corresponds to the change documented in Spanish speaking children with typical development towards the end of their first year in primary school (Rosas et al., 2011; Escobar and Meneses, 2014), when reading instruction begins in schools nationwide, and which in turn drives the relevant transformation in the developmental trajectory of working memory, as previous studies with typically developing children have shown (Demoulin and Kolinsky, 2016). Various authors have suggested that working memory is a weakness in the Down syndrome cognitive performance profile, with an atypical trajectory whose achievements are more tardy (Brock and Jarrold, 2005; Lanfranchi et al., 2012). One might think that, as a function of the bidirectional relation between these two dimensions (Peng et al., 2018), the delayed appearance of these precursors within the evaluated group is related with the documented developmental delay of working memory.

We were also surprised to find no significant correlations among the three phonological awareness tasks assessed, which may seem counterintuitive given that the three tasks tap into the same underlying ability. Phonological awareness is a broad term that includes the ability to identify and manipulate sounds in language (Yopp and Yopp, 2009), but it entails tasks and levels of manipulation that vary in their complexity along a continuum. For example, identifying rhymes is easier than segmenting or blending syllables, and segmenting syllables is easier than segmenting sounds. This may explain why these tasks are not significantly correlated. On the other hand, our sample shows that children’s ability to discriminate final syllables (FSD) and to segment syllables (SS) increased with age and these skills were significantly correlated with print-related skills such as grapheme recognition (GR) and letter name knowledge (KLN), which illustrates the contribution of phonological awareness to the acquisition of the alphabetic principle (Sulzby and Teale, 1991) that is necessary for word reading.

One element from the correlations matrix that stands out is the relations between vocabulary and the other dimensions explored. There is ample documentation of the role which vocabulary plays as a predictor for decoding and phonological awareness, for children with typical development and Down syndrome alike, among native English speakers (Lonigan, 2007; Van Bysterveldt et al., 2010). This finding makes sense when considering previously reported facts and suggests a similar pattern even in the presence of languages with different levels of orthographic transparency. Since it has been reported that vocabulary can be a causal variable for reading development among children with Down syndrome who are native English speakers (Carr, 2000; Laws and Gunn, 2002), future longitudinal studies should observe this relation in Spanish-speaking children.

Another relevant finding is the correlation between syllable segmentation, a skill particular to phonological awareness, and the variables explored in the recognition of the written word and language comprehension. This finding offers evidence favoring the hypothesis that phonological awareness is also important for reading development in children with Down syndrome (Fletcher and Buckley, 2002; Lemons and Fuchs, 2010b), although preliminary studies described its absence (Cossu et al., 1993; Evans, 1994). This result is particularly important in an educational context where it is necessary to review official recommendations to use global teaching methods for reading among children with Down syndrome.

This study has various limitations. First, it is an exploratory study with a sample which is considered to be a “good size” according to international parameters (Dunst and Hamby, 2012; Bunster, 2021), but which would doubtlessly benefit from expanding the number of participants so as to achieve greater analytical potency. Second, no measurements were taken for reading speed and reading pseudo-words, which are considered central skills for reading evaluation. Third, given the size of the analysis groups, it was not possible to perform regression analyses to establish the weight of the variables studied on some measures such as word reading. Fourth, given that it was a cross-sectional study it is not possible to establish a causal relation between the variables, and the trajectory analysis was incomplete. Future studies should have a longitudinal perspective in order to carry out predictive studies regarding reading development and better understand the developmental trajectories of reading in Down syndrome. Fifth, another limitation is that the phonological awareness tasks addressed only syllable discrimination and awareness, and not phoneme awareness. Future studies should include a more profound assessment of phonological awareness skills. Another relevant limitation is that the three groups are not equivalent as the older age group has fewer participants than the other two groups and includes only 10-year-olds, while the other two groups include children across 2 years of development. Finally, all measures in our study are experimental, which limits the extent to which our findings can be generalized. The inclusion of standardized measures of reading precursors and abilities in future research is necessary.

The results obtained allow us to suggest that the reading skills of a group of children with Down syndrome who are native Spanish speakers are sensitive to change and improve with age. The trend observed between age groups suggests that there is an underlying developmental trajectory similar to that described in children with typical development, an affirmation which should be contrasted in future studies. If similar projections are proved, and according to what previous studies have suggested (Lemons and Fuchs, 2010a,b; Baylis and Snowling, 2011; Burgoyne et al., 2012), it will be relevant to incorporate phonological awareness stimulation on the daily practice of teaching children with Down syndrome to read, as well as targeted instruction in other relevant reading precursors such as vocabulary and letter knowledge.

## Data availability statement

The raw data supporting the conclusions of this article will be made available by the authors, without undue reservation.

## Ethics statement

The studies involving human participants were reviewed and approved by Comité de ética de Ciencias Sociales, Pontificia Universidad Católica de Chile. Written informed consent to participate in this study was provided by the participants’ legal guardian/next of kin.

## Author contributions

MT, PA, JE, and RR contributed to conception and design of the study. AA performed the statistical analysis. MT, PA, and JE wrote the first draft of the manuscript. AA and PO wrote sections of the manuscript. All authors contributed to the article and approved the submitted version.

## Funding

MT thanks the support of Agencia Nacional de Investigación y Desarrollo (ANID) vía Fondecyt Regular 1221400. PA thanks the support of Agencia Nacional de Investigación y Desarrollo (ANID) vía Fondecyt Regular 1221349. AA thanks the support of the ANID Millennium Science Initiative Program (ICS2019_024).

## Conflict of interest

The authors declare that the research was conducted in the absence of any commercial or financial relationships that could be construed as a potential conflict of interest.

## Publisher’s note

All claims expressed in this article are solely those of the authors and do not necessarily represent those of their affiliated organizations, or those of the publisher, the editors and the reviewers. Any product that may be evaluated in this article, or claim that may be made by its manufacturer, is not guaranteed or endorsed by the publisher.

## References

[ref1] Abello A. Arango P. S. Rosas Díaz R. (2014). A study of the relationship between musical perception skills and phonological awareness in Spanish/Estudio de la relación entre habilidades de percepción musical y conciencia fonológica en el español. Estud. Psicol. 35, 662–672. doi: 10.1080/02109395.2014.965452

[ref2] Acarlar F. Ege P. Turan F. (2002). Türk çocuklarında üstdil becerilerinin gelişimi ve okuma ile ilişkisi [development of metalinguistic abilities and its relationship with Reading in Turkish children]. Türk Psikoloji Dergisi 15, 9–15.

[ref3] Acevedo M. A. (1993). Development of Spanish consonants in preschool children. J. Childhood Commun. Disord. 15, 9–15. doi: 10.1177/152574019301500202

[ref4] Adams M. J. (1990). Beginning to read. Cambridge: MIT Press.

[ref5] Aguilar Villagrán M. Marchena Consejero E. Navarro Guzmán J. I. Menacho Jiménez I. Alcalde Cuevas C. (2011). Niveles de dificultad de la conciencia fonológica y aprendizaje lector. Revista de Logopedia, Foniatría y Audiología 31, 96–105. doi: 10.1016/S0214-4603(11)70177-2

[ref6] American Educational Research Association, American Psychological Association and National Council on Measurement in Education. (2014). The standards for educational and psychological testing. Washington, DC: AERA Publications Sales.

[ref7] Anderson R. C. Freebody P. (1981). “Vocabulary Knowledge” in Comprehension and teaching: Research reviews. ed. Guthrie J. T. (Newar, Delaware: International Reading Association), 77–117.

[ref9] Bakker A. Cai J. English L. Kaiser G. Mesa V. van Dooren W. (2019). Beyond small, medium, or large: points of consideration when interpreting effect sizes. Educ. Stud. Math. 102, 1–8. doi: 10.1007/s10649-019-09908-4

[ref10] Baylis P. Snowling M. J. (2011). Evaluation of a phonological reading programme for children with down syndrome. Child Lang. Teach. Therapy 28, 39–56. doi: 10.1177/0265659011414277

[ref11] Beck I. L. Juel C. (1995). The role of decoding in learning to read. Am. Educ. 19, 21–25.

[ref12] Bianco M. Pellenq C. Lambert E. Bressoux P. Lima L. Doyen A. L. (2012). Impact of early code-skill and oral-comprehension training on reading achievement in first grade. J. Res. Read. 35, 427–455. doi: 10.1111/j.1467-9817.2010.01479.x

[ref13] Boudreau D. M. (2002). Literacy skills in children and adolescents with down syndrome. Read. Writ. 15, 497–525. doi: 10.1023/A:1016389317827

[ref14] Boudreau D. M. Chapman R. S. (2000). The relationship between event representation and linguistic skill in narratives of children and adolescents with down syndrome. J. Speech Lang. Hear. Res. 43, 1146–1159. doi: 10.1044/jslhr.4305.1146, PMID: 11063236

[ref15] Bravo Valdivieso L. (2002). La conciencia fonológica como una zona de desarrollo próximo para el aprendizaje inicial de la lectura. Estudios pedagógicos 28, 165–177. doi: 10.4067/s0718-07052002000100010

[ref16] Bravo-Valdivieso L. Escobar J. P. (2014). How transparent is Spanish orthography? / ¿Cuán transparente es nuestra ortografía castellana? Estud. Psicol. 35, 442–449. doi: 10.1080/02109395.2014.965455

[ref17] Brock J. Jarrold C. (2005). Serial order reconstruction in down syndrome: evidence for a selective deficit in verbal short-term memory. J. Child Psychol. Psychiatry Allied Discip. 46, 304–316. doi: 10.1111/j.1469-7610.2004.00352.x, PMID: 15755306

[ref18] Bunster J. (2021). Adaptive behaviours in children with down syndrome: A cross-sectional study of developmental trajectories. J. Intellect. Develop. Disabil. 47, 276–286. doi: 10.3109/13668250.2021.1976008 39818575

[ref19] Burgoyne K. Duff F. J. Clarke P. J. Buckley S. Snowling M. J. Hulme C. (2012). Efficacy of a reading and language intervention for children with down syndrome: a randomized controlled trial. J. Child Psychol. Psychiatry 53, 1044–1053. doi: 10.1111/j.1469-7610.2012.02557.x, PMID: 22533801 PMC3470928

[ref20] Cáceres Zuñiga M. F. (2018). Vocabulario receptivo en estudiantes de preescolar en la comunidad de Talca, Chile. Innovación Educativa (México, DF) 18, 193–208.

[ref21] Cadavid-Ruiz N. Quijano-Martínez M. C. Escobar P. Rosas R. Tenorio M. (2016). Validación de una prueba computarizada de lectura inicial en niños escolares colombianos. Ocnos: Revista de Estudios sobre lectura 15, 98–109. doi: 10.18239/ocnos_2016.15.2.1121

[ref22] Caravolas M. Volín J. Hulme C. (2005). Phoneme awareness is a key component of alphabetic literacy skills in consistent and inconsistent orthographies: evidence from Czech and English children. J. Exp. Child Psychol. 92, 107–139. doi: 10.1016/j.jecp.2005.04.003, PMID: 16038927

[ref23] Carr J. (2000). Intellectual and daily living skills of 30-year-olds with Down’s syndrome: continuation of a longitudinal study. J. Appl. Res. Intellect. Disabil. 13, 1–16. doi: 10.1046/j.1468-3148.2000.00003.x

[ref24] Checa E. Galeote M. Soto P. (2016). The composition of early vocabulary in Spanish children with down syndrome and their peers with typical development. Am. J. Speech Lang. Pathol. 25, 605–619. doi: 10.1044/2016_AJSLP-15-0095, PMID: 27893086

[ref25] Cohen J. (1992). Statistical power analysis. Curr. Dir. Psychol. Sci. 1, 98–101. doi: 10.1111/1467-8721.EP10768783/ASSET/1467-8721.EP10768783.FP.PNG_V03

[ref26] Cologon K. (2013). Debunking myths: Reading development in children with down syndrome. Australian J. Teacher Educ. 38, 142–163. doi: 10.14221/ajte.2013v38n3.10

[ref27] Cologon K. Cupples L. Wyver S. (2011). ‘Effects of targeted reading instruction on phonological awareness and phonic decoding in children with down syndrome’, American journal on. Intellect. Dev. Disabil. 116, 111–129. doi: 10.1352/1944-7558-116.2.111, PMID: 21381947

[ref28] Coltheart M. (2006). Dual route and connectionist models of reading: an overview. Lond. Rev. Educ. 4, 5–17. doi: 10.1080/13603110600574322

[ref29] Cossu G. Marshall J. C. (1990). Are cognitive skills a prerequisite for learning to read and write? Cogn. Neuropsychol. 7, 21–40. doi: 10.1080/02643299008253433

[ref30] Cossu G. Rossini F. Marshall J. C. (1993). When reading is acquired but phonemic awareness is not: a study of literacy in Down’s syndrome. Cognition 46, 129–138. doi: 10.1016/0010-0277(93)90016-O 8432093

[ref31] Cupples L. Iacono T. (2000). Phonological awareness and Oral Reading skill in children with down syndrome. J. Speech Lang. Hear. Res. 43, 595–608. doi: 10.1044/jslhr.4303.595, PMID: 10877431

[ref32] Cupples L. Iacono T. (2002). The efficacy of “whole word” versus “analytic” reading instruction for children with down syndrome. Read. Writ. Interdiscip. J. 15, 549–574. doi: 10.1023/A:1016385114848

[ref33] de Graaf G. Buckley F. Skotko B. G. (2016). Estimation of the number of people with down syndrome in the United States. Genet. Med. 19, 439–447. doi: 10.1038/gim.2016.127, PMID: 27608174

[ref34] de la Cruz Paulino M. (2017). Dificultades de aprendizaje: estrategias para la enseñanza de la lectoescritura en niños con síndrome de down. Revista Internacional PEI: Por la Psicología y Educación Integral 13, 1–24.

[ref35] Defior S. (2008). ¿Cómo facilitar el aprendizaje inicial de la lectoescritura? Papel de las habilidades fonológicas. Infancia y aprendizaje 31, 333–345. doi: 10.1174/021037008785702983

[ref36] Defior S. Serrano F. (2014). Diachronic and synchronic aspects of Spanish: The relationship with literacy acquisition/Aspectos diacrónicos y sincrónicos del español: relación con la adquisición del lenguaje escrito. Estud. Psicol. 35, 450–475. doi: 10.1080/02109395.2014.974422

[ref37] Dehaene S. Cohen L. (2011). The unique role of the visual word form area in reading. Trends Cogn. Sci. 15, 254–262. doi: 10.1016/J.TICS.2011.04.003, PMID: 21592844

[ref38] Demoulin C. Kolinsky R. (2016). ‘Does learning to read shape verbal working memory?’, Psychonomic bulletin and review. Springer New York LLC 23, 703–722. doi: 10.3758/s13423-015-0956-7, PMID: 26438254

[ref39] Duff F. J. Reen G. Plunkett K. Nation K. (2015). Do infant vocabulary skills predict school-age language and literacy outcomes? J. Child Psychol. Psychiatry 56, 848–856. doi: 10.1111/jcpp.12378, PMID: 25557322 PMC4674965

[ref40] Dunn L. M. Dunn L. M. (1997). The Peabody picture vocabulary test, Third. Circle Pines, MN: American Guidance Sevice.

[ref41] Dunst C. J. Hamby D. W. (2012). Guide for calculating and interpreting effect sizes and confidence intervals in intellectual and developmental disability research studies. J. Intellect. Develop. Disabil. 37, 89–99. doi: 10.3109/13668250.2012.673575, PMID: 22530580

[ref42] Eberhard D. Simons G. Fennig C. (2022). Ethnologue: Languages of the world. Twenty-fif. Dallas, Texas: SIL International.

[ref44] Ehri L. C. Nunes S. R. Willows D. M. Schuster B. V. Yaghoub-Zadeh Z. Shanahan T. (2001). Phonemic awareness instruction helps children learn to read: evidence from the National Reading Panel’s meta-analysis. Read. Res. Q. 36, 250–287. doi: 10.1598/rrq.36.3.2

[ref45] Escobar J. P. Meneses A. (2014). Initial reading predictors in Spanish according to SES: Is semi-transparency sufficient to explain performance?/Predictores de la lectura inicial en español según NSE: ¿es suficiente la semi-transparencia Para explicar su desempeño? Estud. Psicol. 35, 625–635. doi: 10.1080/02109395.2014.965458

[ref46] Evans R. (1994). Phonological awareness in children with down syndrome. Down Syndrome Res. Practice 2, 102–105. doi: 10.3104/reports.38

[ref47] Fletcher H. Buckley S. (2002). Phonological awareness in children with down syndrome. Downs Syndr. Res. Pract. 8, 11–18. doi: 10.3104/reports.123 11915432

[ref48] Florit E. Roch M. Altoè G. Levorato M. C. (2009). Listening comprehension in preschoolers: the role of memory. Br. J. Dev. Psychol. 27, 935–951. doi: 10.1348/026151008X397189, PMID: 19994487

[ref50] Galeote M. Sebastián E. Checa E. Rey R. Soto P. (2011). The development of vocabulary in Spanish children with down syndrome: comprehension, production, and gestures. J. Intellect. Develop. Disabil. 36, 184–196. doi: 10.3109/13668250.2011.599317, PMID: 21843033

[ref52] Galeote M. Soto P. Sebastián E. Rey R. Checa E. (2012). La adquisición del vocabulario en niños con síndrome de Down: datos normativos y tendencias de desarrollo. Infancia y Aprendizaje 35, 111–122. doi: 10.1174/021037012798977502

[ref53] Goetz K. Hulme C. Brigstocke S. Carroll J. M. Nasir L. Snowling M. (2008). Training reading and phoneme awareness skills in children with down syndrome. Read. Writ. 21, 395–412. doi: 10.1007/s11145-007-9089-3

[ref54] Goikoetxea E. (2006). Reading errors in first-and second-grade readers of a shallow orthography: evidence from Spanish. Br. J. Educ. Psychol. 76, 333–350. doi: 10.1348/000709905X52490, PMID: 16719967

[ref55] Goswami U. Bryant P. (2016). Phonological skills and learning to read. New York, NY: Routledge. 10.1111/j.1749-6632.1993.tb22977.x 8323121

[ref57] Hernández Salazar V. Talou C. (2005). “Alfabetización inicial en niños con Síndrome de Down” in XII Jornadas de Investigación y Primer Encuentro de Investigadores en Psicología del Mercosur. Buenos Aires: Facultad de Psicología - Universidad de Buenos Aires, 261–263.

[ref58] Herrera L. Defior S. (2005). Una Aproximación al Procesamiento Fonológico de los Niños Prelectores: Conciencia Fonológica, Memoria Verbal a Corto Plazo y Denominación. Psykhe (Santiago) 14, 81–95. doi: 10.4067/s0718-22282005000200007

[ref59] Hessling Prahl A. Jones R. Melanie Schuele C. Camarata S. (2022). Phonological awareness intervention using a standard treatment protocol for individuals with down syndrome. Child Lang. Teach. Therapy 38, 22–42. doi: 10.1177/02656590211033013/ASSET/IMAGES/LARGE/10.1177_02656590211033013-FIG1.JPEG

[ref01] Hoff E. (2003). The specificity of environmental influence: Socioeconomic status affects early vocabulary development via maternal speech. Child Development 74, 1368–1378. doi: 10.1111/1467-8624.00612 14552403

[ref60] Hulme C. Goetz K. Brigstocke S. Nash H. M. Lervåg A. Snowling M. J. (2012). The growth of reading skills in children with down syndrome. Dev. Sci. 15, 320–329. doi: 10.1111/j.1467-7687.2011.01129.x, PMID: 22490173

[ref62] Jiménez D. P. Flórez-Romero R. (2013). ¿La lectura y la literatura como derechos? El caso de la discapacidad intelectual. Revista de la Facultad de Medicina 61, 175–184.

[ref64] Kalaycı G. Ö. Diken Ö. (2022). Relations between the levels of fluent Reading and Reading comprehension and the levels of phonological awareness of individuals with down syndrome in Turkey. Int. J. Disabil. Dev. Educ. 69, 707–721. doi: 10.1080/1034912X.2020.1727419

[ref65] Kay-Raining Bird E. Cleave P. L. McConnell L. (2000). Reading and phonological awareness in children with down syndrome: a longitudinal study. Am. J. Speech Lang. Pathol. 9, 319–330. doi: 10.1044/1058-0360.0904.319

[ref66] Kennedy E. Flynn M. (2003). Early phonological awareness and reading skills in children with down syndrome. Down Syndrome Res. Practice 8, 100–109. doi: 10.3104/reports.136, PMID: 14502837

[ref68] King S. A. (2020). Reading instruction for children with down syndrome: Extending research on behavioral phenotype aligned interventions. Exceptionality 30, 92–108. doi: 10.1080/09362835.2020.1749631

[ref69] King S. Rodgers D. Lemons C. J. (2022). The effect of supplemental Reading instruction on fluency outcomes for children with down syndrome: a closer look at curriculum-based measures. Except. Child. 88, 421–441. doi: 10.1177/00144029221081006/ASSET/IMAGES/LARGE/10.1177_00144029221081006-FIG1.JPEG

[ref70] Lanfranchi S. Baddeley A. Gathercole S. Vianello R. (2012). Working memory in down syndrome: is there a dual task deficit? J. Intellect. Disabil. Res. 56, 157–166. doi: 10.1111/j.1365-2788.2011.01444.x, PMID: 21726323

[ref71] Language and Reading Research Consortium (2017). Oral language and listening comprehension: same or different constructs? J. Speech Lang. Hear. Res. 60, 1273–1284. doi: 10.1044/2017_JSLHR-L-16-0039 28475679

[ref72] Laws G. Gunn D. (2002). Relationships between reading, phonological skills and language development in individuals with down syndrome: a five year follow-up study. Read. Writ. 15, 527–548. doi: 10.1023/A:1016364126817

[ref73] Lemons C. (2015). Adapting phonological awareness interventions for children with down syndrome based on the behavioral phenotype: a promising approach? Intellect. Dev. Disabil. 53, 271–288. doi: 10.1352/1934-9556-53.4.271, PMID: 26214557

[ref74] Lemons C. J. Fuchs D. (2010a). Modeling response to Reading intervention in children with down syndrome: an examination of predictors of differential growth. Read. Res. Q. 45, 134–168. doi: 10.1598/RRQ.45.2.1

[ref75] Lemons C. J. Fuchs D. (2010b). Phonological awareness of children with down syndrome: its role in learning to read and the effectiveness of related interventions. Res. Develop. Disabilities. Pergamon 31, 316–330. doi: 10.1016/j.ridd.2009.11.002, PMID: 19945821

[ref76] Lervåg A. Hulme C. Melby-Lervåg M. (2018). Unpicking the developmental relationship between Oral language skills and Reading comprehension: It’s simple, but complex. Child Dev. 89, 1821–1838. doi: 10.1111/cdev.12861, PMID: 28605008

[ref02] Lonigan C. J. (2007). “Vocabulary development and the development of phonological awareness skills in preschool children,” in Vocabulary Aquisition: implications for Reading Comprehension. eds. Wagner R. K. Muse A. E. Tannenbaum K. R. (The Guilford Press), 15–31.

[ref77] Mariscal S. López-Ornat S. Gallego C. Gallo P. Karousou A. Martínez M. (2007). Evaluation of communicative and linguistic development using the Spanish version of the MacArthur-bates inventories. Psicothema 19, 190–197. PMID: 17425886

[ref78] Míguez-Álvarez C. Cuevas-Alonso M. Saavedra Á. (2022). Relationships between phonological awareness and Reading in Spanish: a meta-analysis. Lang. Learn. 72, 113–157. doi: 10.1111/LANG.12471

[ref79] Mimeau C. Ricketts J. Deacon S. H. (2018). The role of orthographic and semantic learning in word Reading and Reading comprehension. Sci. Stud. Read. 22, 384–400. doi: 10.1080/10888438.2018.1464575

[ref80] Ministerio de Educación. (2008). Palabras + palabras aprendamos a leer: Manual para las y los Docentes. Santiago, Chile: Ministerio de Educación.

[ref81] Morales M. F. Farkas C. Aristotelous E. MacBeth A. (2020). The impact of contextual. Maternal and Prenatal Factors on Receptive Language in a Chilean Longitudinal Birth Cohort’, Child Psychiatry and Human Develop. 52, 1106–1117. doi: 10.1007/s10578-020-01091-5, PMID: 33130923 PMC8528774

[ref82] Næss K.-A. B. (2016). Development of phonological awareness in down syndrome: a meta-analysis and empirical study. Dev. Psychol. 52, 177–190. doi: 10.1037/a0039840, PMID: 26689762

[ref83] Næss K.-A. B. Melby-Lervåg M. Hulme C. Lyster S. A. H. (2012). Reading skills in children with down syndrome: a meta-analytic review. Res. Dev. Disabil. 33, 737–747. doi: 10.1016/j.ridd.2011.09.019, PMID: 22115916

[ref84] Nash H. Heath J. (2011). The role of vocabulary, working memory and inference making ability in reading comprehension in down syndrome. Res. Dev. Disabil. 32, 1782–1791. doi: 10.1016/J.RIDD.2011.03.007, PMID: 21536407

[ref85] National Reading Panel (US), National Institute of Child Health and Human Development (US). (2000). Teaching children to read: An evidence-based assessment of the scientific research literature on reading and its implications for reading instruction: Reports of the subgroups. National Institute of Child Health and Human Development, National Institutes of Health.

[ref86] Peng P. Barnes M. Wang C. C. Wang W. Li S. Swanson H. L.. (2018). Meta-analysis on the relation between reading and working memory. Psychol. Bull. 144, 48–76. doi: 10.1037/BUL0000124, PMID: 29083201

[ref87] Quinn J. M. Wagner R. K. Petscher Y. Lopez D. (2015). Developmental relations between vocabulary knowledge and Reading comprehension: a latent change score modeling study. Child Dev. 86, 159–175. doi: 10.1111/CDEV.12292, PMID: 25201552 PMC4331220

[ref88] R Core Team. (2022). ‘R: A language and environment for statistical computing’. Vienna, Austria: R Foundation for Statistical Computing.

[ref89] Revelle W. (2020). ‘Psych: Procedures for psychological, psychometric, and personality research’. Evanston, Illinois.

[ref90] Ricci L. A. (2011). Exploration of reading interest and emergent literacy skills. Int. J. Special Educ. 26, 80–91.

[ref91] Roch M. Florit E. Levorato C. (2015). Follow-up study on reading comprehension in Down’s syndrome: the role of reading skills and listening comprehension. Int. J. Lang. Commun. Disord. 46, 231–242. doi: 10.3109/13682822.2010.487882, PMID: 21401820

[ref92] Rosas R. (2013). ABCDeti: Test de la competencia lectora inicial. Manual de aplicación. Santiago de Chile: Pontificia Univeridad Católica de Chile.

[ref93] Rosas R. Escobar J. P. Ramírez M. P. Meneses A. Guajardo A. (2017). Impact of a computer-based intervention in Chilean children at risk of manifesting reading difficulties. Infancia y Aprendizaje J. Study of Educ. Develop.: J. Study of Educ. Develop. 40, 158–188. doi: 10.1080/02103702.2016.1263451

[ref94] Rosas R.. (2011). ‘Construcción y validación de una prueba de evaluación de competencia lectora inicial basada en computador’, Pensamiento Educativo. Revista de Investigación Latinoamericana (PEL) 48, 43–62. doi: 10.7764/PEL.48.1.2011.4

[ref95] Rupley W. H. (2012). Building conceptual understanding through vocabulary instruction. Reading Horizons: J. Literacy and Language Arts 51:51

[ref96] Scarborough H. (2002). “Connecting early language and literacy to later Reading (dis)abilities: evidence, theory, and practice” in Handbook of early literacy research. eds. Neuman S. B. Dickinson D. K. (New York: The Guilford Press), 97–110.

[ref97] Seymour P. H. K. Aro M. Erskine J. M. collaboration with COST Action A8 network (2003). Foundation literacy acquisition in European orthographies. Br. J. Psychol. 94, 143–174. doi: 10.1348/000712603321661859, PMID: 12803812

[ref98] Share D. L. (1995). Phonological recoding and self-teaching: sine qua non of reading acquisition. Cognition 55, 151–218. doi: 10.1016/0010-0277(94)00645-2, PMID: 7789090

[ref99] Sherman S. L. Allen E. G. Bean L. H. Freeman S. B. (2007). Epidemiology of down syndrome. Ment. Retard. Dev. Disabil. Res. Rev. 13, 221–227. doi: 10.1002/mrdd.20157 17910090

[ref101] Snowling M. J. Hulme C. Mercer R. C. (2002). A deficit in rime awareness in children with down syndrome. Read. Writ. Interdiscip. J. 15, 471–495. doi: 10.1023/A:1016333021708

[ref102] Solheim O. J. Frijters J. C. Lundetræ K. Uppstad P. H. (2018). Effectiveness of an early reading intervention in a semi-transparent orthography: a group randomised controlled trial. Learn. Instr. 58, 65–79. doi: 10.1016/J.LEARNINSTRUC.2018.05.004

[ref103] Strasser K. Larraín A. López de Lérida S. Lissi M. R. (2010). La Comprensión Narrativa en Edad Preescolar: Un Instrumento para su Medición. Psykhe (Santiago) 19, 75–87. doi: 10.4067/S0718-22282010000100006

[ref104] Subsecretaría de Educación Parvularia, G. de C. (2018). Bases Curriculares: Educación Parvularia. Santiago de Chile: Gobierno de Chile.

[ref105] Subsecretaría de Educación Parvularia, G. de C. (2021). Informe de caracterización de la educación parvularia 2020: Descripción estadística del sistema educativo asociado al nivel de educación parvularia en Chile. Santiago. Available at: moz-extension://58ca7099-00dc-bd42-9ff9-5ff60fdec0bc/enhanced-reader.html?openApp&pdf=https%3A%2F%2Fparvularia.mineduc.cl%2Fwp-content%2Fuploads%2F2021%2F07%2FInforme_EP_cierre_2020.pdf (Accessed: 28 December 2022).

[ref106] Sulzby E. Teale W. (1991). “Emergent literacy” in Handbook of reading research. ed. Pearson P. D. (UK: Routledge), 727–757.

[ref107] Tannenbaum K. R. Torgesen J. K. Wagner R. K. (2009). Relationships between word knowledge and Reading comprehension in third-grade children. Sci. Stud. Read. 10, 381–398. doi: 10.1207/S1532799XSSR1004_3

[ref108] Tenorio M. (2016). La Mesita: Escritorio virtual para la estimulación del desarrollo lector de niñas y niños con Síndrome de Down. Manual de Trabajo. Santiago, Chile: Centro UC Tecnologías de Inclusión CEDETi.

[ref109] Torgerson C. Brooks G. Hall J. (2006). A systematic review of the research literature on the use of phonics in the teaching of reading and spelling. Nottingham: DfES Publications.

[ref110] Torgesen J. K. Wagner R. K. Rashotte C. A. Burgess S. Hecht S. (1997). Contributions of phonological awareness and rapid automatic naming ability to the growth of word-Reading skills in second-to fifth-grade children. Sci. Stud. Read. 1, 161–185. doi: 10.1207/s1532799xssr0102_4

[ref111] Troncoso M. V. Flórez J. (2011). Comprensión en la lectura de las personas con síndrome de Down. Revista Síndrome de Down 109, 50–59.

[ref112] van Bysterveldt A. Gillon G. (2014). A descriptive study examining phonological awareness and literacy development in children with down syndrome. Folia Phoniatr. Logop. 66, 48–57. doi: 10.1159/000364864, PMID: 25472792

[ref113] van Bysterveldt A. Van Gillon G. Foster-cohen S. (2010). ‘Literacy environments for children with down syndrome: What’s happening at home?’, Down Syndrome Res. Practice, 12, pp. 98–102. doi: 10.3104/reports.2111

[ref114] Van Bysterveldt A. K. Gillon G. T. Moran C. (2007). Enhancing phonological awareness and letter knowledge in preschool children with down syndrome. Int. J. Disabil. Dev. Educ. 53, 301–329. doi: 10.1080/10349120600847706

[ref115] Verucci L. Menghini D. Vicari S. (2006). Reading skills and phonological awareness acquisition in down syndrome. J. Intellect. Disabil. Res. 50, 477–491. doi: 10.1111/j.1365-2788.2006.00793.x, PMID: 16774633

[ref116] Vieiro-Iglesias P. Fernández-Garrido Á. Gómez-Taibo M. L. García-Real T. (2015). Los fonemas, las sílabas y los morfemas como unidades de procesamiento ortográfico en el proceso lector de alumnos de Educación Infantil. Revista de Estudios e Investigación en Psicología y Educación 9, 023–028. doi: 10.17979/reipe.2015.0.09.254

[ref118] Whitehurst G. J. Lonigan C. J. (1998). Child development and emergent literacy. Child Dev. 69, 848–872. doi: 10.1111/J.1467-8624.1998.TB06247.X 9680688

[ref119] Wickham H. Averick M. Bryan J. Chang W. McGowan L. François R.. (2019). Welcome to the {tidyverse}. J. Open Source Software 4:1686. doi: 10.21105/joss.01686

[ref120] Yopp H. K. Yopp R. H. (2009). Phonological awareness is child’s play. Young Child. 64, 12–21.

[ref121] Ziegler J. C. Goswami U. (2005). Reading acquisition, developmental dyslexia, and skilled Reading across languages: a psycholinguistic grain size theory. Psychol. Bull. 131, 3–29. doi: 10.1037/0033-2909.131.1.3, PMID: 15631549

